# Approaching Retinal Ganglion Cell Modeling and FPGA Implementation for Robotics

**DOI:** 10.3390/e20060475

**Published:** 2018-06-19

**Authors:** Alejandro Linares-Barranco, Hongjie Liu, Antonio Rios-Navarro, Francisco Gomez-Rodriguez, Diederik P. Moeys, Tobi Delbruck

**Affiliations:** 1 Robotic and Technology of Computers Lab, University of Seville, ES41012 Sevilla, Spain; 2 Institute of Neuroinformatics, ETHZ-UZH, CH8057 Zurich, Switzerland

**Keywords:** neuromorphic engineering, event-based processing, Address-Event-Representation, Dynamic Vision Sensor, approach sensitivity cell, Retina Ganglion Cell, robotic, FPGA

## Abstract

Taking inspiration from biology to solve engineering problems using the organizing principles of biological neural computation is the aim of the field of neuromorphic engineering. This field has demonstrated success in sensor based applications (vision and audition) as well as in cognition and actuators. This paper is focused on mimicking the approaching detection functionality of the retina that is computed by one type of Retinal Ganglion Cell (RGC) and its application to robotics. These RGCs transmit action potentials when an expanding object is detected. In this work we compare the software and hardware logic FPGA implementations of this approaching function and the hardware latency when applied to robots, as an attention/reaction mechanism. The visual input for these cells comes from an asynchronous event-driven Dynamic Vision Sensor, which leads to an end-to-end event based processing system. The software model has been developed in Java, and computed with an average processing time per event of 370 ns on a NUC embedded computer. The output firing rate for an approaching object depends on the cell parameters that represent the needed number of input events to reach the firing threshold. For the hardware implementation, on a Spartan 6 FPGA, the processing time is reduced to 160 ns/event with the clock running at 50 MHz. The entropy has been calculated to demonstrate that the system is not totally deterministic in response to approaching objects because of several bioinspired characteristics. It has been measured that a Summit XL mobile robot can react to an approaching object in 90 ms, which can be used as an attentional mechanism. This is faster than similar event-based approaches in robotics and equivalent to human reaction latencies to visual stimulus.

## 1. Introduction

The biological retina is the extension of the brain that perceives visual information. Visual processing begins when photons stimulate the light-sensitive photo-receptor rod and cone cells in the retina. The activity of several rod cells is received by bipolar cells in a second neuron layers. Bipolar cells are in charge of detecting luminosity changes. These cells convert the information into electrical signals and send them through intermediate networked layers of cells to 15–20 types of retinal ganglion cells. They perform visual processing before visual information arrives to the visual cortex in the brain. In the central nervous system, high priority information arrives first to the brain so it is processed in a higher priority, or even in an involuntary and reflexive way.

In [1], Muench et al. identified a ganglion cell type in the mouse retina, they called it the approach sensitivity cell (AC), as it is sensitive to the approaching motion of objects (expanding objects). The detection of approaching motion elicits behaviors such as startle and protective motor responses in animals and humans. These responses are also important to predict collisions. This kind of function is also required in autonomous vehicles and robotics for obstacle detection and avoidance, which is one of the most important tasks in autonomous vehicles and mobile robots’ environment perception. This task is basically carried out using range sensors or computer vision.

Range sensors are based on flight time, the most used are ultrasonic range sensors (sonars), 2D and 3D laser range sensors (LIDAR) and structured light vision sensors (such as Microsoft Kinect). With all of them obstacles can be detected in a range of few millimeters to several meters and in a field of view of up to 360°, in the case of 3D LIDAR.

Computer vision is based on the computation of the information (frames) captured by CCD cameras, and its goal is to get salient information. To detect objects in the robot pathway, two approaches are possible: the use of a single camera or two cameras in a stereo vision system. In both cases several processes that consume a considerable amount of resources must be carried out, such as: (1) camera calibration: for lens distortion correction; (2) image preprocessing: for image damage restoration and non-useful information elimination; (3) image processing: for salient information extraction, e.g., binarization, segmentation, features extraction and objects identification; and (4) in the case of stereo vision, correspondence search, epipolar rectification and triangulation are necessary for distance estimation. These processes must be done for all captured images in real time, which poses a very high computational cost. Conventional digital cameras capture 25 frames per second (fps) implying an inter-frame-time of 40 ms, which is usually too short to perform the needed operations. In [2], Nam et al. compared LRF and RGB solutions for SLAM, obtaining a maximum computation speed of 27 and four frames-per-second, respectively (37 and 250 ms) without the inclusion of the robot latency. In [3], Akolkar et al. state that low rate spikes processing from frame-based sensors can lose up to 70% of the information.

The Dynamic Vision Sensor [4] (DVS) mimics the temporal dynamic responses of the retina by asynchronously outputting events signaling brightness changes, like the bipolar cells. Every pixel works independently from others in such a way that when the detected brightness (log intensity) changes by more than a preset threshold from the pixel’s memorized value of brightness, a spike is produced by that pixel in the sensor output. The communication protocol between event based sensors and other neuromorphic hardware is called Address Event Representation (AER). The AER protocol encodes the x–y address of the pixel, plus a polarity, where the temporal change has surpassed the threshold and it transmits that address using an asynchronous handshake protocol. This polarity is represented with one bit that indicates whether the change of brightness was positive or negative. The use of digital cameras to emulate retinal ganglion cells functionality demands an enormous amount of computational resources to extract that crucial information from the frames in the available inter-frame time, as can be extracted from the computational model presented in [5] by Wei et al. The DVS pixel implements the functionality of the ON and OFF transient bipolar cell. Other ganglion cell functionalities, such as the local and global motion detection and approach responses, are suitable to be implemented both in software (jAER; see jAER Open Source Project for real time sensory-motor processing for event-based sensors and systems http://www.jaerproject.org/) and hardware (EU VISUALISE project; see http://www.visualise-project.eu/). For hard real-time systems this biological mimicking technique can be crucial and the development of visual embedded systems that are able to perform relatively complex visual tasks, like fast object detection and tracking, is critical, as we evaluate in this paper.

There are many promising applications in the literature that take advantage of this event-based processing concept, such as in [6] (one of the first) where DVS sensor output was connected to several convolution event-based chips in parallel to detect particular shapes, followed by winner-take-all filters, to make it possible to move motors in order to follow one particular object in real time with sub-millisecond visual processing latencies. In [7], a spiking neural network is implemented in SpiNNaker [8] for DVS event processing to drive a mobile robot in a cognitive way. In [9], a neuro-inspired motor controller is modeled in the spike-based domain to drive a robotic head to follow an object detected by a DVS sensor and a set of event-based object trackers [10]. According to [11], human latency for arm reaction from visual stimuli is 100 ms, and it is longer for legs. In [12], Clady et al. reported a time-to-contact algorithm in this kind of event-based applications and sensors [13]. In this work the algorithm was tested in a robotic application for obstacle avoidance. A latency per event of 20 μs and a processing time of 330 ms were reported for the robot in motion. In [14], an attentional event-based algorithm—called EVA that needs 1 ms in the best case plus 15 μs of the DVS latency—was integrated and tested on the iCub robot and it was compared to classic computer vision equivalent algorithms, which need 50–60 ms plus the camera latency to obtain a photograph, which requires another 30 ms. In this case, the motor actuation delay is not included. In this work, we characterized a bio-inspired way of detecting approaching objects with lower latencies, but also with more restrictions on the visual input, because of the use of DVS. Then, we applied that to a robotic platform to measure the latency of the reflexive movement of the robot when an approaching object is detected.

Entropy is normally used in information systems to demonstrate the wellness of a distribution. There are several works related to event-based processing where the entropy is used in formulations and demonstrations in this sense. For example, Peng & Zhao et al. in [15] used entropy to demonstrate the Bag Of Events (BOE) concept as a feature extraction method, based on probability theory; in [16], Belbachir et al. demonstrate how well a set of events for a block of time can detect a falling person for health care applications; and, among others, in [17], Mishra et al. used entropy to validate classifications of groups of spikes produced for objects tracking.

This paper is an extension of first reports [18] and we provide more detailed explanations of the methods, new results, an entropy study that measures how deterministic the output of the system is, and its application to mobile robots. It is structured as follows. The next section shows the results of the implemented AC biological model both in software and hardware, including an entropy analysis, and its application to a mobile robot. Section 3 presents discussions. Then, Section 4 presents the material and methods of this work that explains (1) the biological model of the AC; (2) the software implementation of the model in Java; for the open-source jAER project; (3) the hardware implementation of an AC on a field programmable gate array (FPGA) using a set of AER platforms; and (4) the integration of the system in the Summit XL robot governed by the Robotic Operating System (ROS). Finally, Section 5 presents the conclusions.

## 2. Results

This section presents the results of the biological model implemented in software and hardware, which is presented in details in further sections, and a comparison between its application in a robotic scenario and biological responses.

### 2.1. Software Model

**Figure 1:**
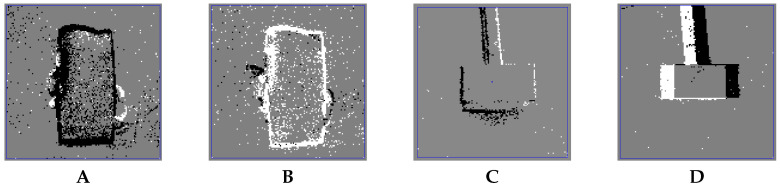
jAER histogram for (**A**): an approaching object, (**B**): a receding object, (**C**): a left motion object and (**D**): a right motion object. Black and white dots represent the excitatory (OFF) and inhibitory (ON) events respectively of DVS silicon retina accumulated for 40 ms.

**Figure 2:**
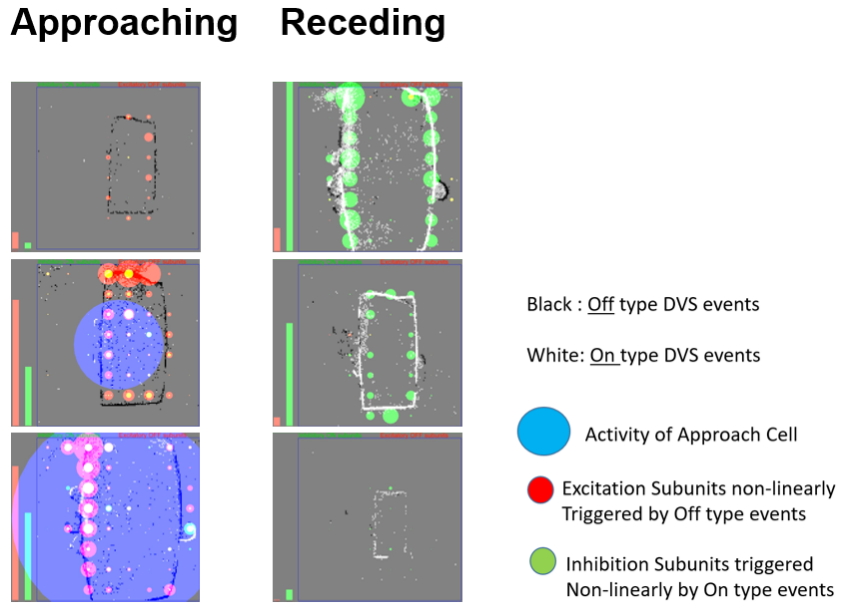
Result of AC software model implemented in jAER. Red dots represents OFF subunits activity, which is the excitatory AC activity. Green dots represent ON subunits activity, which is the inhibitory AC activity. The blue circle represents the AC software output firing rate.

Figure 1 shows a histogram of a DVS silicon retina capturing an approaching/receding motion (A,B) and a lateral movement (C,D) of two objects in jAER. jAER is an open-source JAVA software framework that takes a stream of input events (from the hardware through USB or from a stored file) and a histogram of them in the screen for a configured period of time, allowing anyone to program their own event-based processing method in the code and to represent results of it in the screen. In Figure 2, the output of the DVS sensor is represented with black and white pixels (OFF and ON events), while the activity of the AC subunits is represented with red and green circles. Left bars in the figures summarize the OFF (red bar) and ON (green bar) activity for all the subunits. When these bars are at the same height or when the green ones are higher, the AC membrane potential is not enough to make the cell to fire, as it happens in the left figure due to a right to left movement. In this case, the subunits that compose the response of the AC are accumulating the same number of OFF and ON events, canceling the excitatory (red) and inhibitory (green) activity. Therefore, there is no detection of an expanding or approaching object. On the other hand, only when the OFF activity (excitatory red bar) is stronger than the ON activity (inhibitory green bar), the AC fires. This situation is represented with a blue circle in the right figure for the software AC model.

### 2.2. Hadware Model

**Figure 3:**
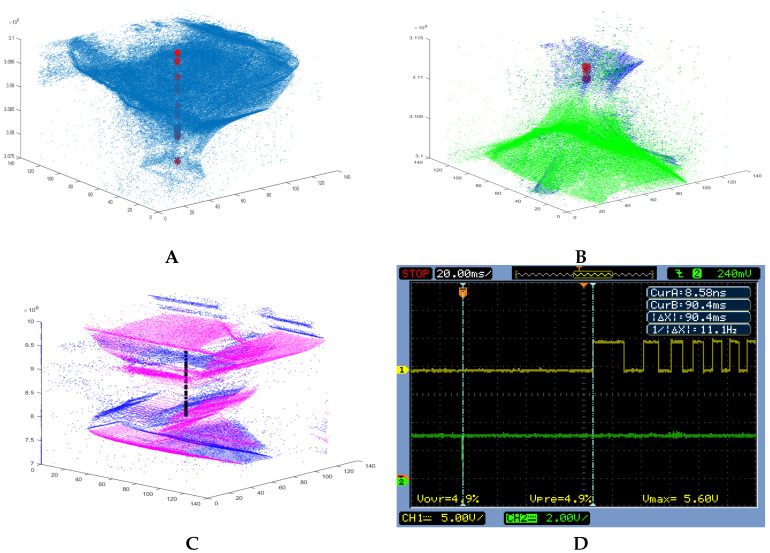
(**A**,**B**): Result of AC hardware implementation displayed in a 3D visualization of address-event and the AC firing for stimuli like Figure 1A. Red stars show the hardware AC firing, blue and green dots represent the excitatory (OFF) and inhibitory (ON) events respectively. (**A**): shows an approaching object and (**B**): shows a receding object that starts approaching at the end of the graph. (**C**): black dots represent the AC-HW output, which is active when the object is approaching. Magenta and blue represent OFF and ON events respectively. The bottom part corresponds to lateral movement (no AC output), and the top part corresponds to approaching movement (with AC output). In (**D**): oscilloscope screen shot showing the latency between the AC output spike and the hall sensor of the front-left wheel first edge, where 90.4 ms represents the maximum latency.

Figure 3 shows space-time views of the event data of both the output of the DVS and the output coming from the FPGA AC-HW implementation. The vertical dimension is the increasing timestamp, while the horizontal dimensions are the (x, y) addresses of the pixel array. In the top left part (A), the object was small (too far) in the beginning and it expands (approaches) along time, producing output spikes of the AC-HW cell (red stars). In (B), as time increases, the area of the green (ON)/blue (OFF) dots (events) first decreases and then increases, indicating that the object first recedes (shrinks in the view) and then approaches (expands in the view). It is shown that the AC-HW fires when the object approaches and it is silent when recedes. In (C), a combined experiment is shown using the stimulus represented in Figure 1C,D. That stimulus contains an object with lateral and approaching/receding movements. Also, (C) shows a space-time view of the input and output of the AC-HW with a lateral movement in the bottom part (low timestamps), followed by an approaching movement of the object in top part of the figure (higher timestamps).

The minimum measured inter-spike-interval (ISI) for each AC-HW firing signal is 16.6 ms and the maximum is 75.7 ms. The average ISI is 31.2 ms. These latencies depends on the model parameters for a particular scenario, as we explain in the materials section. If the decay factor is more frequent due to denser input event stream, then the latency is smaller. Therefore, these latencies are application dependent, and the parameters have to be adjusted properly for the target application. A dynamic adaptation of the parameters according to the input event bandwidth would be very convenient.

In hardware, the latency to process each incoming event depends on the state machine. For a 50 MHz clock of the FPGA, the latency is 160 ns per event. This latency is the time difference between the input request of a new event to the AC input, and the output request of an output event of the AC. It was measured using ChipScope embedded in the FPGA. Table 1 shows the comparison between the software and hardware performances in terms of latency and power. The software performance was measured in two types of PCs with very different resources. The software latency was measured by using the “measure filter performance” option in jAER. Here, the latency represents the average for the stream of processed events.

We have applied this hardware model to a real robotic experiment in which it was used as an attentional mechanism for obstacle avoidance. The experiment set up is described in the material section in detail. It consists of a DVS retina connected to a platform where the AC-HW runs on FPGA. When the AC fires, a micro-controller acts as an interface between the neuron and the robotic operating system (ROS) running in the embedded PC of the Summit XL mobile robot.

Figure 3D shows a scope screen shot measuring the latency of the system when reacting to the detection of an approaching object. Channel 1 is connected to the hall sensor of the front left wheel, and channel 2 is connected to the REQ signal of the AC-HW output. For each output spike of the AC, the Summit XL is commanded to go backward 10 cm. The latency measured from the AC spike to the start of the movement of the wheel is 90.4 ms in the worst case. This time is several ms smaller since it is more suitable to start the movement in the middle of a pulse of the hall sensor output. It was also measured that the delay between the AC spike and the USB command of the microcontroller to the Summit XL is 10 μs.

### 2.3. Entropy Analysis

In order to study how deterministic the response of this AC is, an entropy analysis was carried out. The AC-HW was stimulated with the continuous repetition of a small recording composed of the events represented in Figure 1A (approaching) followed by the ones comprised in Figure 1B (receding). In this case, it was expected to obtain AC output events only for those parts of the stimulus where the object is approaching and no events when the object is receding. In order to determine this fact through time, the entropy of the system was calculated for a repetitive experiment. The entropy is defined as in Equation (1):

(1) $$H=-\sum p_{i}*log\left(p_{i}\right),\mathrm{where}\sum p_{i}=1$$

For each iteration (i) of the stimulus, $AC_{e}(i)$ is the number of events the AC-HW is spiking, $AC_{eap}(i)$ is the number of events the AC spikes while the object is approaching and $AC_{ere}(i)$ the number of events the AC spikes while the object is receding (if any). Therefore, $AC_{e}(i)=AC_{eap}(i)+AC_{ere}(i)$ for each iteration of the stimulus. For the whole recording, expression (2) is obeyed.

(2) $$\sum AC_{eap}\left(i\right)+\sum AC_{ere}\left(i\right)=\sum AC_{e}\left(i\right)\Rightarrow \sum \frac{AC_{e}\left(i\right)-AC_{ere}\left(i\right)}{\sum AC_{eap}\left(i\right)}=1\Rightarrow p_{i}=\frac{AC_{e}\left(i\right)-AC_{ere}\left(i\right)}{\sum AC_{eap}\left(i\right)}$$

For a recording of 20 iterations of the approaching/receding recording, an entropy H=2.77 is obtained, with log(20) = 2.99 being the maximum value of H, which represents the best deterministic behavior of the AC model.

In general terms, the model is not totally deterministic because of the non-linearities of the model imposed by the decay factor and the subunits models, as it happens in biology. It is also important to consider that the initial state of the AC at the beginning of each iteration of the stimulus is not the same, which provokes different responses.

## 3. Discussion

In the biological retina, the detection of an approaching object is computed there before the arrival of the visual information to the visual cortex in the brain. This mechanism reduces considerably latencies for reflexive movements. In classic computer vision, obstacle avoidance is usually not based on cameras because of the imposed delay they have and the high computational cost needed to process the visual information to make a decision. When a camera is used in robotics it has to take photographs in a continuous way and the system has to process these frames in the inter-frame-time, which usually is 40 ms. Nevertheless, the time consumed from the visual stimulus to the start of processing its corresponding frame is also around 40 ms. These computer vision systems process the visual information in a pipeline and parallel way, when possible, in order to reduce these latencies. As it can be seen, there is a big difference between biological and classical computer vision systems. Thanks to the design of specialized cameras that reproduce the behavior of some cells in the retina, it is possible to mimic these biological solutions. For the particular case presented in this work, these specialized cameras reproduce the ON and OFF activity of the retinas (bipolar cells), while the approaching sensitivity is computed by another type of cell in the retina, called retina-ganglion cells. With these sensors available, the RGC model can be computed in the event-based domain. The division of the sensor resolution in a set of clusters distributed in a matrix allows to easily discriminate lateral movements, as is shown in Section 4.2 (Figure 6). These clusters of pixels are computed in subunits taking into account the OFF and ON accumulation of events. In this paper, we assumed that a DVS retina would fire OFF events along the contour of an object when this is approaching. Obviously this depends on luminosity, thus the opposite could happen. Nevertheless, this issue is solved by considering an opposite polarity and checking that the number of OFF subunits is increasing along time. This polarity parameter, among other parameters, can be modified in real time through jAER for both the software and hardware implementations. In the hardware case, the parameters are sent through USB to the FPGA registers allowing to adjust the AC firing rate, the OFF threshold, the synaptic weight, the firing mode (IF or Poisson), the ON and OFF weights ratio, the surround suppression activation, the decay time of the neuron and the number of subunits to be computed. Once these parameters are properly adjusted for the particular experiment, the AC can be used in robot navigation for obstacle detection when they approach to the vision sensor. Taking into account the latency from the visual stimulus to the AC output, which was reported to be 16.6 ms as minimum in a previous section; the reaction latency should be 100 ms, which is near the human arm reaction [11], and improved with respect to previous works [12,14].

## 4. Material and Methods

### 4.1. The Approach Sensitivity Cell Biological Model

**Figure 4:**
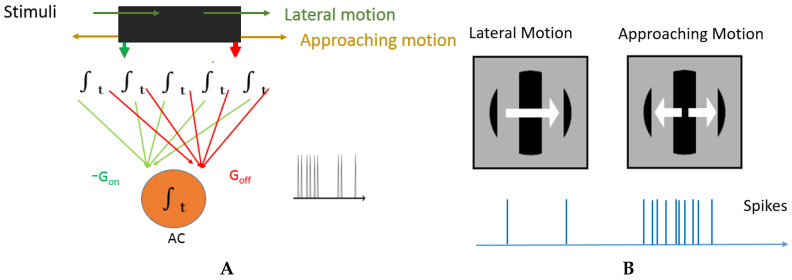
(**A**): Biological model of AC: green lines are inhibitory activity from subunits and red lines correspond to excitatory activity. AC fires if excitatory activity is stronger; (**B**): Lateral movements produces similar Gon and Goff activity while approaching produces stronger Goff.

In [1], it is reported that the AC receives excitatory and inhibitory inputs from small subunits. The excitatory inputs are from the so called OFF type subunits which respond to the decrease of brightness, and the inhibitory inputs are from the so called ON type subunits which respond to the increase of brightness. The ON and OFF type subunits cancel out each other when there is lateral motion. To be sensitive to approaching motion, the crucial point is that the cell **nonlinearly** integrates the potential of a broad area of ON–OFF subunits in its receptive field. The nonlinearity takes the form of an expansive function with a finite threshold. The thresholded responses of the subunits are summed into the AC (see Figure 4A). Due to this nonlinearity, weak global inhibitions will not cancel out local strong excitations, since the latter have stronger impact.

The synaptic input to the AC is calculated as a weighted subtraction of the total ON and OFF units as in (3):

(3) $$I_{net}=G_{off}*\sum V_{off}-G_{on}*\sum V_{on}$$

The membrane potential of the AC is calculated as (4):

(4) $$dV_{mem}=I_{net}*dT$$

where dT is the inter-spike interval. To calculate the input of each subunit, the potential of each subunit is half rectified to perform non-linearity as described in [1].

### 4.2. AC Software Implementation

**Figure 5:**
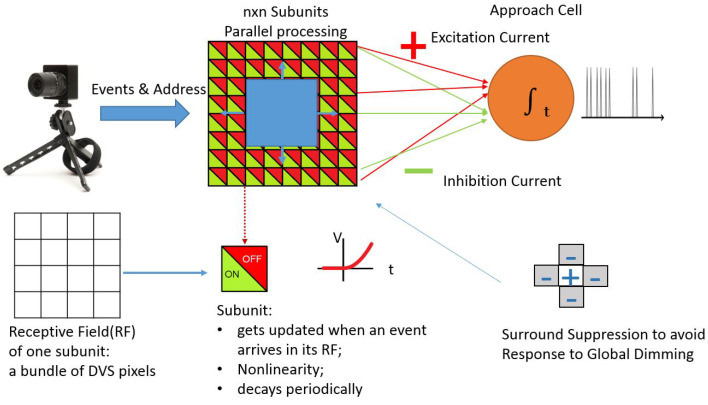
Software implementation of the Approach Sensitivity Cell.

Figure 5 shows the software model of the AC. One AC was designed to have nxn subunits that process the input events in their receptive field in parallel. Each subunit has ON and OFF parts received from mxm pixels (called receptive field (RF) in Figure 5) for a DVS128 sensor that has 128 × 128 pixels. Therefore, nxm = 128. Whenever an event with certain polarity (ON or OFF) is received in the receptive field of one subunit, the membrane potential of the ON or OFF subunit is updated. A threshold-linear nonlinearity is implemented for each subunit. All the subunits decay simultaneously and periodically. The decay time constant can be set to be tuned to a particular motion speed. The membrane potentials of all the OFF subunits are summed to provide the excitation current to the AC; the potentials of all the ON subunits are summed to provide the inhibition current to the AC. An ON center-OFF surround subtract scheme is implemented to avoid the AC firing from global dimming of the environment. In this scheme when a subunit accumulates excitation current, it starts to inhibit the four principal neighbors (bottom-right part of the figure).

The potential of the center cell is calculated as in Equation (5).

(5) $$V_{centertoAC}=V_{center}-\frac{\sum V_{surround}}{n}$$

where *n* is the number of surrounding cells of the center cell; *n* can be 0, 2, 3 or 4 depending on whether it is on the center, boarder or corner of the receptive field.

**Figure 6:**
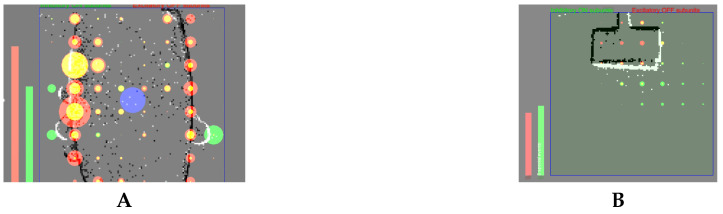
Java software model output of subunits and AC firing. (**A**): AC fires when object approaches (**B**): AC is silent when object movement is lateral (bottom right to top left diagonal movement in this case). The red-yellow disk shows the OFF excitation while the green disk shows the ON inhibition. There are 8 × 8 subunits in this case and each subunit process the activity of 16 × 16 pixels.

The membrane potential of the AC is calculated as in (4). It is compared either with a preset threshold voltage or a randomly generated number when Poisson firing mode is enabled. The AC fires when it is larger than the threshold at integrate-and-fire mode or a random number at Possion-fire mode. Important parameters for the AC Java model are the ON/OFF weight ratio, and the excitation strength. A parameter is also set for the maximum firing rate of the AC. Figure 6 shows the software implementation subunits states of the AC in jAER. The object is a dark smart-phone. The phone is moved closer to the camera, causing it to apparently expand. The result corresponds to the working principle of the AC. The AC fires (blue circle in the middle) when the phone approaches (Figure 6A), because there are more OFF events generated than ON events as the light intensity decreases on the expanding border, and this causes a majority of excitatory subunits (red/yellow circles). The AC is actively inhibited when the phone recedes, and the ON and OFF activities are balanced when it moves laterally (Figure 6B). Green circles represent the inhibitory activity of the subunits. These subunits are clusters of pixels.

### 4.3. AC Hardware Implementation

**Figure 7:**
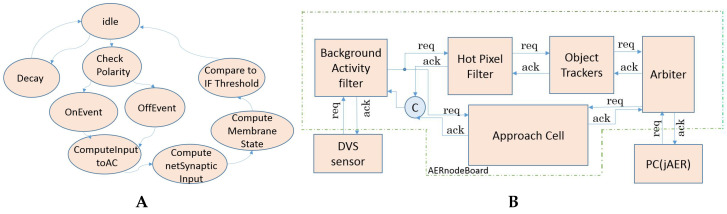
Approach Sensitivity Cell hardware: (**A**): State Machine. (**B**): FPGA architecture.

Figure 7A shows the state machine of the AC. The right branch shows the main state transition. Once receiving an event, after the potential of the subunit is updated (*OnEvent* or *OffEvent*), the input current of each subunit is calculated (*ComputeInputtoAC*). Then, the net current of all the subunits is computed (*ComputenetSynapticInput*). After receiving the input current, the AC updates its membrane potential state following the integrate-and-fire neuron model (*ComputeMembranState*). Then the membrane potential of the AC is compared to the threshold (*ComparetoIFThreshold*) to determine whether it should fire or not. The cell then goes back to *idle* state. The left branch of the state machine is the decay scheme. A global counter counts the time lapsed. When the counter overflows, all the subunits decay by a shared programmable factor (*Decay*). If there is no event received, the cell stays in *idle* state.

In [10], direct hardware integration of architectures for digital post-processing of DVS retina output was presented. The algorithms have the potential of lightweight, power-efficient, and fast retina processing that can be efficiently embedded inside a camera. This work reported the synthesis of a set of a background activity filter, a mask filter and four object trackers described in VHDL, in a Lattice FPGA platform, in order to be used for demonstrators. This work was used as a starting point to integrate vision neural models, like [19], with the implementation of the Object Motion Cell functionality of the Retina Ganglion Cells. It is again used here for the AC. Figure 7B shows a block diagram of the hardware. DVS retina output is connected to the hardware. These sensors usually have some low frequency activity in the pixels. In order to remove this activity to avoid wrong behaviors in the next processing blocks, a background activity filter is implemented. Basically, this filter is parameterized to remove pixels activity that has low frequency and is not related to neighbors activity. The output of this filter is split in two ways, one for the tracker and the other one for the AC-HW model. The trackers way includes a second filter, called hot-pixel filters, which can remove high frequency pixels activity. Then the object tracker is basically a neuron model based on a state machine and a small memory able to increase its membrane potential if the activity of a cluster of pixels in the DVS is higher than a configurable threshold. This neuron model is able to follow the detected cluster along the visual field of the DVS if the activity is present. If no activity is detected for a period of time, the neuron is reset. The output of this object tracker neuron is a stream of AER, which addresses represent the center of mass of the tracked object. In this hardware up to four tracking neurons are implemented in cascade in such a way that up to four different objects can be tracked in the scene without collision between them. The output of the hardware is combined using an arbiter. This arbiter is merging the event stream coming from the trackers and the AC.

The AC is described with the VHDL hardware description language and validated with simulations using Xilinx ISE 4.7 design tool. Then it was implemented in the Spartan6 1500LXT FPGA for the AERNode platform [20]. This AC implementation requires 4.5 K slice registers (2% of the FPGA), 11.2 K slice LUTs (12%) and 2 DSP blocks (1%). The AERNode platform allows multi-board communication with conventional parallel handshake AER chips, serial Low-Voltage Differential Signaling (LVDS) connections or robots control with the adequate motor interfaces. A daughter board based on an OpalKelly module, called OKAERTool [21], is used for monitoring, sequencing, logging or playing events from and to the AERNode board. It is able to sequence events from its on-board DDR2 128 MB SDRAM to the AERNode board and to monitor its output through USB2.0 in jAER. OKAERTool is used for debugging.

**Figure 8:**
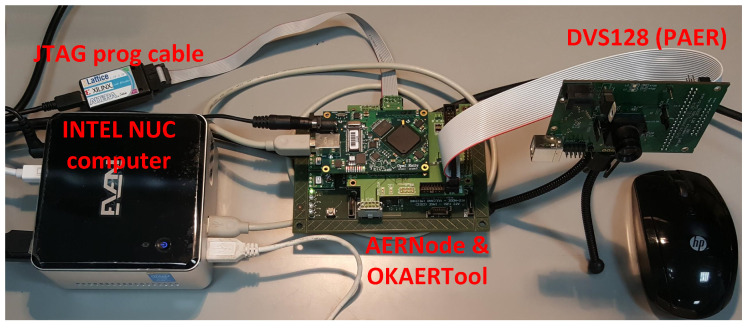
The hardware system setup.

The implementation is structured in two levels. The first level is called Mother Cell (**AC-MC**), which is ready to host one or many AC in different visual fields, together with the circuit to receive the corresponding parameters for each cell through an Serial Peripheral Interface (SPI) to the USB interface microcontroller on the OKAERTool. Figure 8 shows the testing scenario, where a DVS128 retina is connected to the OKAERTool merger. The OKAERtool is plugged to the AERNode, which is running the system showed in Figure 7B. OKAERTool is configured to send to the AERNode the DVS128 output and to collect the output of the AERNode and send it to jAER through USB2.0. The output of the AERNode is configured to be the merge of the DVS128 after a Background-Activity-Filter in the FPGA, and the output of the AC. The USB2.0 output of the OKAERTool is connected to the NUC embedded computer, which is running jAER.

### 4.4. Mobile Robot System Integration

**Figure 9:**
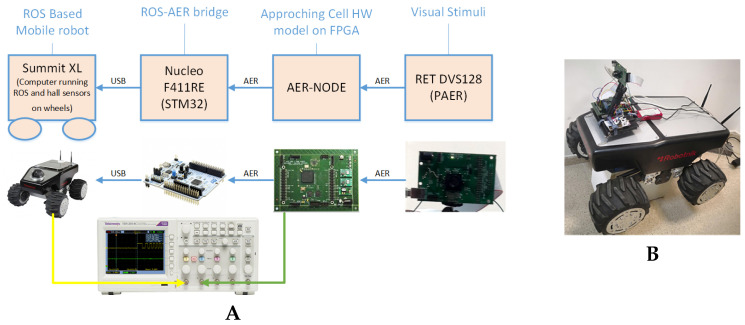
The mobile robot system setup (**A**) and the integration photograph (**B**).

For system validation we have integrated this hardware model in a robotic platform to measure the reaction latency for obstacle avoidance. The robot used is the Summit XL, which is a mobile robot with dimensions of 722 × 613 × 392 mm, 45 kg weight, 3 m/s maximum speed and 5 h autonomy in continuous movement. Each wheel is driven by a 250 W brush-less motor. This robot is governed by an embedded i5 computer that runs the robotic operating system (ROS) under Linux. The wheels are powered by the batteries (LiFePO4 15Ah@24V) through natch bridges commuted through USB interfaces. The robot includes a Wifi router for TCP/IP connection that allows remote control. An USB interface allows to receive movement orders to the wheels through ROS. In this particular scenario (see Figure 9) we have connected the hardware setup explained in the previous subsection to a STM32 micro-controller. This micro controller is a 32 bit MCU that runs a simple C++ code that is waiting for an interrupt produced by AC output spikes. The interrupt is in charge of sending a USB command to the ROS running in the embedded computer. This command will order the movement of the four wheels in parallel. Each wheel has attached a hall encoder sensor to measure the rotation speed. The output of these sensors produces a square signal, whose frequency is related to the speed. In the previous result section it is shown that the latency of the robot, including this AC-HW and AER-USB interface, is bellow 100 ms.

## 5. Conclusions

This paper offers software and FPGA implementation of the AC model for real time detection of expanding dark objects and its application in robotic for obstacle avoidance. The FPGA implementation requires less than 0.8 W power and has a latency of 180 ns, which is smaller than that of the software approach, 370 ns on the embedded “Next Unit of Computing” Intel NUC computer. The software model running on a more powerful desktop PC takes 55 ns to process one event (on average) with a power consumption higher than 100 W. The latency from visual stimulus to motor movement is similar to that measured for humans 100 ms (16.6 ms for visual processing), as demonstrated with the integration of the hardware in a mobile robot platform. Similar state of the art works needed more than two tens of milliseconds for the visual information processing, while event-based solutions considerably reduce visual processing tasks [22]. An entropy analysis of the AC response was performed and it demonstrated that AC response is not totally deterministic because of the non-linearities of the model. Future work will be focused on integrating the AC with the OMC [19] and/or other algorithms where the AC serves as an attention mechanism.

## Figures and Tables

**Table 1:** Performance Table.

	jAER (64-bit Intel NUC, 4 GB RAM, i5-4250U, 1.30 GHz)	jAER (64-bit PC, 16 GB RAM, i7-4770K, 3.50 GHz)	FPGA Xilinx Spartan6, 50 MHz
Latency	370 ns/ev at 0.2 Mev/s, at 5% CPU load	55 ns/ev at 0.2 Mev/s, at 3% CPU load	160 ns/ev at any event rate
Power	6.2 W static, +2.5 W dynamic (running jAER)	160 W	0.775 W static, +0.05 W dynamic (processing events)
